# Early-Stage Prototype Assessment of Cost-Effective Non-Intrusive Wearable Device for Instant Home Fetal Movement and Distress Detection: A Pilot Study

**DOI:** 10.3390/diagnostics14171938

**Published:** 2024-09-02

**Authors:** Hana Mohamed, Suresh Kalum Kathriarachchi, Nipun Shantha Kahatapitiya, Bhagya Nathali Silva, Deshan Kalupahana, Sajith Edirisinghe, Udaya Wijenayake, Naresh Kumar Ravichandran, Ruchire Eranga Wijesinghe

**Affiliations:** 1 Department of Computer Engineering, Faculty of Engineering, University of Sri Jayewardenepura, Nugegoda 10250, Sri Lanka; hanasophie111@gmail.com (H.M.); egt18538@sjp.ac.lk (N.S.K.); deshankalupahana@sjp.ac.lk (D.K.); udayaw@sjp.ac.lk (U.W.); 2 Department of Engineering, Institute of Chemical Research (ICR), Kyoto University, Kyoto 611-0011, Japan; kathriarachchi.sureshkalum.5r@kyoto-u.ac.jp; 3 Department of Information Technology, Faculty of Computing, Sri Lanka Institute of Information Technology, Malabe 10115, Sri Lanka; nathali.s@sliit.lk; 4 Center for Excellence in Informatics, Electronics & Transmission (CIET), Sri Lanka Institute of Information Technology, Malabe 10115, Sri Lanka; 5 Department of Anatomy, Faculty of Medical Sciences, University of Sri Jayewardenepura, Nugegoda 10250, Sri Lanka; edirisinghe@sjp.ac.lk; 6 Center for Scientific Instrumentation, Korea Basic Science Institute, 169-148 Gwahak-ro, Yuseong-gu, Daejeon 34133, Republic of Korea; 7 Department of Electrical and Electronic Engineering, Faculty of Engineering, Sri Lanka Institute of Information Technology, Malabe 10115, Sri Lanka

**Keywords:** fetal phonocardiography (fPCG), fetal distress, fetal heart rate and movement detection, self-applicable home monitoring system

## Abstract

Clinical fetal monitoring devices can only be operated by medical professionals and are overly costly, prone to detrimental false positives, and emit radiation. Thus, highly accurate, easily accessible, simplified, and cost-effective fetal monitoring devices have gained an enormous interest in obstetrics. In this study, a cost-effective and user-friendly wearable home fetal movement and distress detection device is developed and assessed for early-stage design progression by facilitating continuous, comfortable, and non-invasive monitoring of the fetus during the final trimester. The functionality of the developed prototype is mainly based on a microcontroller, a single accelerometer, and a specialized fetal phonocardiography (fPCG) acquisition board with a low-cost microphone. The developed system is capable of identifying fetal movement and monitors fetal heart rhythm owing to its considerable sensitivity. Further, the device includes a Global System for Mobile Communication (GSM)-based alert system for instant distress notifications to the mother, proxy, and emergency services. By incorporating digital signal processing, the system achieves zero false negatives in detecting fetal movements, which was validated against an open-source database. The acquired results clearly substantiated the efficacy of the fPCG acquisition board and alarm system, ensuring the prompt identification of fetal distress.

## 1. Introduction

Losing a baby is devastating, especially during the final trimester when the pregnancy is so close to term. This critically impacts the mother’s health by leading to feelings of distress, guilt, self-blame, depression, and a generally negative well-being. Some of the causes of stillbirths, such as nuchal cords (wrapping of the umbilical cord around the fetal neck), high blood pressure, and preterm and post-term labor [1], can be overcome with immediate medical assistance. The tragedy of fetal death is not experienced uniformly; the stillborn rate varies greatly in countries across the globe. The stillbirth rate (per 1000 births) in Sri Lanka has increased from 6.54% to 6.9% during the past decade [2] despite global technological breakthroughs in remote and home health monitoring. A staggering stillborn rate of 1.9 million was recorded in 2019 alone, making such cases “Neglected Tragedies” [3]. However, it can be reduced with immediate medical assistance.

‘Fetal Distress’ is all-pervasive and is used to indicate suspected fetal hypoxia, where oxygen supply to the fetus is inadequate, leading to alterations in fetal heart rhythm, decreased fetal movement, meconium staining pre-labor, and fetal growth restriction [4]. This non-reassuring fetal status can be detrimental if left undiagnosed. The manifestation of fetal life and well-being depends upon the maternal perception of fetal activity. A healthy fetus is linked with a perception of higher fetal activity during the final trimester [5], while the perception of reduced fetal movement is strongly correlated with fetal death. Reduced fetal movements or signs of fetal distress can go unnoticed by the mother as she perceives fetal movements when she shifts position, even when fetal death is confirmed [6]. A study conducted by Monasta et al. [7] revealed that 72% of Intrauterine Fetal Deaths (IUFDs) occurred above 30 weeks of gestational age and concluded that adoption of evidentiary diagnostic protocols could help in preventing future IUFDs. Moreover, it highlighted the importance of reducing the diagnostic gap between its known and unknown causes, revealing the importance of continuous monitoring. Detection of fetal distress can only be performed through electronic monitoring. In current primary care, cardiotocography (CTG) requires accurate probe placement by a trained professional [8], and the lack of automated analysis and episodic measurement with the necessity for serial clinical visits particularly burdens mothers in rural areas, limiting access to perinatal care.

Numerous studies link CTGs of compromised fetuses to false positive assurances and an inability to prevent catastrophic perinatal events despite increased caesareans [9,10,11,12]. Ultrasound is considered safe, but its recommended selective use [13] raises the need for more frequent and economical fetal health-and-distress monitoring. This need is even greater in home environments. 

**Figure 1:**
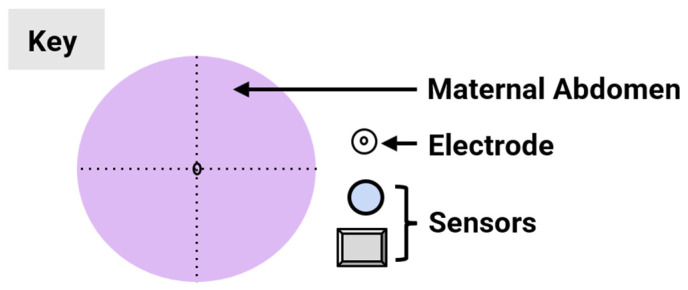
Visual comparison of the number and placement of sensors and electrodes on the maternal abdomen in existing electronic fetal health monitoring devices.

Over the past decade, non-invasive fetal home monitoring methods have become increasingly prevalent, with many devices achieving medical-grade standards, each with its own set of advantages and limitations. Table 1 summarizes and compares the existing fetal monitoring devices and prototypes, focusing on the fetal signal processing algorithm, optimum type, number, and placements of sensors to ensure low complexity, power consumption, cost-effectiveness, ease of operation, portability, high reliability, and comfort of the proposed device. Figure 1 represents the different symbols used to make this comparison.

The Invu system by Mhajna et al. [14] is one system where the measurements of Fetal Heart Rate (FHR) and Maternal Heart Rate (MHR) were significantly correlated with that of the CTG. The Invu system consists of eight passive bio-potential (electric) sensors and four acoustic sensors (microphones) for robust signal acquisition, overcoming variability due to changes in fetal movement and body habitus. While the system succeeded in providing reliable tracings and allowed for self-administration without the need for sensor repositioning, the study only included women who had pregnancies free of pathologies, and monitoring was limited to 32 weeks of gestation to full term. However, as one of the main objectives of the Invu system was accuracy, the increased computational complexity and cost due to the high number of sensors were not factored in. This criterion was instead met by Delay et al. [15], by developing a wearable fetal movement monitoring system in a non-clinical environment using a single accelerometer sensor module containing a triple-axis accelerometer and triple-axis gyroscope. This device was able to realize Fetal Movement (FM) as early as the 26th gestational week and had high accuracy, although not medical grade. Their use of a removable memory chip instead of the wireless Radio Frequency (RF) transmission used in the Invu system posed the advantage of reducing undesirable exposure of energy to the fetus. Lai et al. [16] shared this concern and devised a custom-made inertial measurement unit using an accelerometer complete with its own housing, which contained a removable SD card for offline Analog-to-Digital Conversion (ADC) and inertial signal processing. The device contained a maximum of eight acoustic sensors for real-time monitoring and discrimination of fetal movement by the 32nd (average) gestational week, but the occurrence of a false positive rate due to the extreme sensitivity of fetal movements posed difficulties in consistent, accurate detection. This led the authors to conclude that while only transmitting, wearable sensors can accurately detect fetal breathing movements, other FMs, such as startles, can be precisely detected with their proposed sensor system and signal analysis algorithm, even during maternal activity.

For healthy, late pregnancies (42 gestational weeks), Khandoker et al. [20] proposed a 4-channel fetal phonocardiogram (fPCG), which employed four piezoelectric vibrational (acoustic) sensors placed equidistant from the maternal navel that was able to record the fPCG regardless of fetal position, although the device seemed bulky and required the mother to remain in a sitting position, which limits patient movement and comfort. A combination of fPCG and fECG monitoring methods is seen in the works of Yuan et al. [17], where a base ECG collector containing a processor, signal acquisition unit, Bluetooth, and low power supply module was placed at the center of the maternal abdomen, forming a 3-Lead (channel) using five AgCl electrodes. The device also conveyed warnings wirelessly to an Android™ smartphone when an abnormal fetal heart rate was detected. However, placement of the ECG collector on the maternal abdomen could pose risks of overheating and hurting the patient. Further, for the rural communities in Africa where electronic fetal monitoring was previously unheard of, Boeing et al. [21] proposed an inexpensive and user-friendly fetoscope. In a similar vein, Wei et al. [22] and Hema et al. [23] used a fetoscope resembling a microphone sensor and a digital stethoscope, respectively, for the monitoring of FHR. The former used a single microphone sensor with a wireless signal acquisition system, while the latter incorporated the digital stethoscope with an electromyography/microelectromechanical system (EMG/MEMS) sensor for cancellation of the maternal electrocardiogram (mECG) signal. 

The choice of sensor is critical in determining the components of a signal that will be acquired. This importance has driven advancements in sensor technology, and recently, substantial growth has been made in the utilization of fiber-optic-based sensors [24,25]. Abeywardhana et al. [18] employed fetal photoplethysmography (fPPG) through optical fiber sensors for the sensing of FHR. While the sensors had minimal weight, size, and cost and were more sensitive to FM than maternal perception, their use of a high pass filter removed smaller FM signals, and sensor distortion made FM harder to perceive. Yang et al. [19] conducted a pilot study on the use of inertial sensors for the measurement of fetal sternal seismocardiography (fSCG) and fetal gyrocardiography (fGCG) and found that, through the use of accelerometers and gyroscopes, FHR can be accurately monitored with results being comparable to fetal cardiotocography (fCTG) but not fetal electrocardiography (fECG), which was found to be more accurate.

In this study, an early-stage prototype assessment for a cost-effective, non-intrusive wearable device for instant home fetal movement and distress detection is presented. Given the device’s early development stage, all testing was performed using data from a publicly available fetal movement database [26], supplemented by simulation testing. Several studies demonstrate the viability of using open-source datasets for conducting fetal movement analysis [27,28,29]. This negated the need for conducting clinical testing on pregnant mothers to conduct a feasibility analysis, thus not requiring ethical clearance, as the study did not directly involve human subjects. The feasibility results of the study suggest a promising potential for preventing IUFD and reducing diagnostic gaps and fetal abnormalities. Additionally, the device enhances fetal survival, especially in areas with limited hospital access, while promoting higher compliance with prenatal care and alleviating maternal psychological stress. The cost-effectiveness of the design ensures accessibility to continuous monitoring for diverse socio-economic backgrounds, minimizing hospital visits and associated expenses. This approach is particularly reassuring for financially constrained expecting mothers, reducing transport costs and ensuring peace of mind with continuous at-home monitoring.

## 2. Materials and Methods

### 2.1. The Operational Algorithm

**Figure 2:**
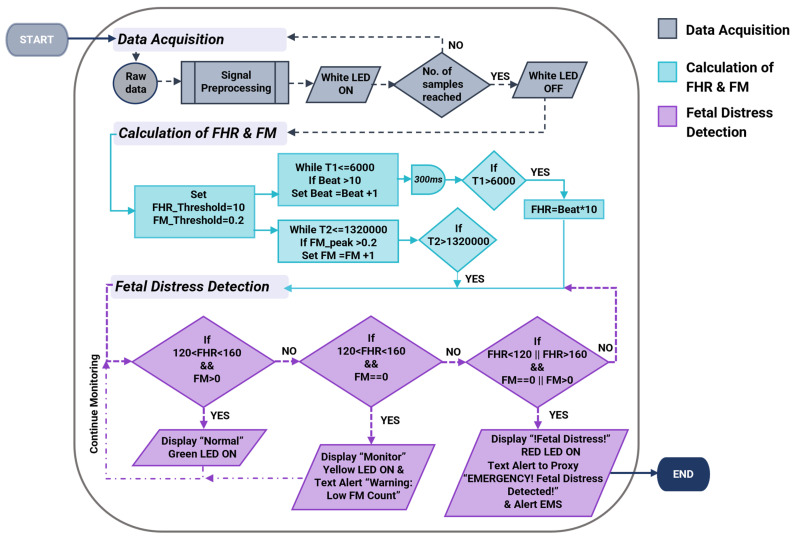
The flowchart illustrates the design algorithm for the cost-effective, non-intrusive wearable device for instant home fetal movement and distress detection.

The operational process begins with the setup and initialization of serial communication as well as acquiring data from the two sensors through signal pre-processing (Figure 2). 

Upon the completion of data acquisition and filtering of the two signals, the algorithm calculates Fetal Heart Rate (FHR) and Fetal Movement (FM). The FHR calculation incorporates a heart rate count within 6 s and multiplied by a factor of 10 to obtain the average heartbeat in beats per minute (bpm). The delay of 300 milliseconds (ms) in between each beat calculation allows sufficient time for the MCU to prevent doubling the heartbeat. FM is counted when an amplitude greater than 0.2 AMU (around 0.015–0.06 g [28]) is identified. Fetal distress is detected when the values obtained for FHR and FM deviate from the ground truth. The ground truth of FHR lies between 120–160 bpm [30], while the period of fetal rest, where no fetal movement is expected, lies between 22–75 min [31], which corresponds to a maximum of 4500 s. Based on the algorithm, if the FHR is within the normal range and sufficient fetal movements are observed, there are no concerns of risk. However, if fetal movement remains absent for more than 4500 s and FHR < 120 bpm or FHR > 160 bpm, it indicates a non-reassuring state. Accordingly, the LCD and LEDs of the system will represent the current fetal status depending on these variables. Upon identifying fetal distress, the system initiates the alarm mode and contacts emergency services.

The final stage of the operational process involves configuring the system of the proposed device by defining the different inputs, outputs and processes undertaken by the microcontroller unit.

### 2.2. System Configuration

**Figure 3:**
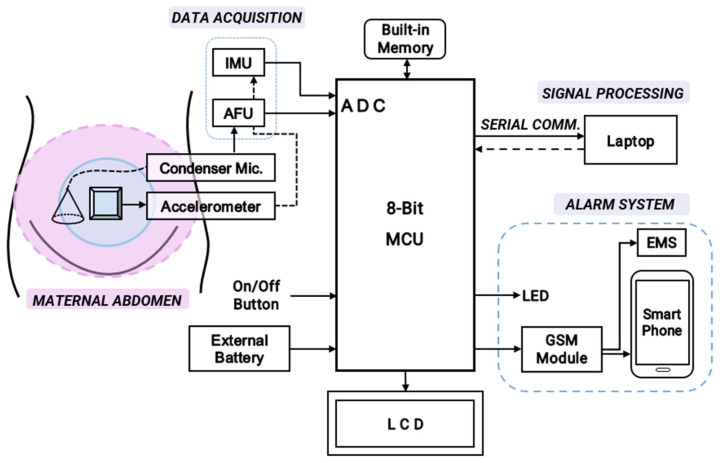
The overall schematic diagram for hardware implementation of the cost-effective, non-intrusive wearable device for instant home fetal movement and distress detection.

Figure 3 presents the overall block diagram for the development of the cost-effective, non-intrusive wearable device for instant home fetal movement and distress detection prior to its hardware implementation.

The device consists of a single digital MPU6050 accelerometer (InvenSense, Shenzhen, China) and a sensitive CA0106 electret condenser microphone (KINGWEI, Shenzhen, China). Fetal kicks are most frequent in the middle of the maternal abdomen. Hence, the accelerometer is placed in the middle of the maternal abdomen to count fetal movements (FMs). The condenser microphone, which is capable of detecting FHR frequencies around 20–200 Hz [12], is placed slightly lower to record the fetal heart rate (FHR). The analog output from the electret is first pre-processed using an Amplifier Filter Unit (AFU) for data acquisition and consequently sent to the control unit (an 8-bit Arduino UNO). Raw accelerometer data from an open-source database are processed using a laptop and fed to the fetal distress detection algorithm. A 16 × 2 LCD interfaced with an I2C module is used to display the FHR and FM count. The alarm system consists of LEDs that alert the patient at home, while Emergency Services (EMS) and the smartphone of a proxy contact are alerted using a power-efficient SIM800L GSM module (Yixing Micro Technology, Shenzhen, China). To initiate/halt the monitoring process, an on/off button is used. For long-term, portable recording, an external battery can be utilized. According to the system configuration (Figure 3), the first step for obtaining the inputs involves the analog acquisition of the fPCG signal to detect fetal heart rate. Digital signal processing is then used to acquire the fetal movement (acceleration) signal to count fetal movements.

### 2.3. Development of the Fetal Phonocardiography (fPCG) Acquisition Board

**Figure 4:**
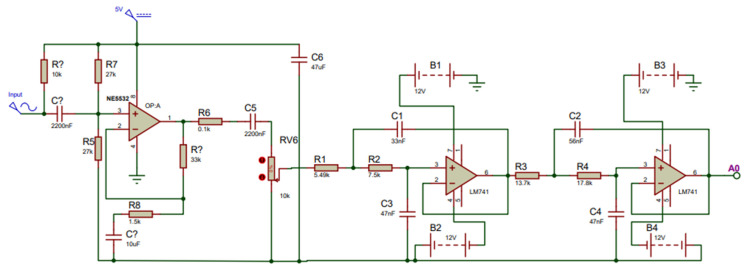
Simulation setup for fetal phonocardiography (fPCG) acquisition circuit, consisting of a preamplifier and fourth-order Butterworth filter.

When developing the fPCG acquisition board, fail-safe conventional design principles were followed. Prior to the signal transmission towards the MCU, the signal of the condenser microphone must be pre-amplified to increase the amplitude of the fetal heart to a voltage that can be detected by the MCU. To construct the amplifier, the Op-Amp NE5532 (Texas Instruments, Dallas, TX, USA) was used due to its 60 dB superior open gain and requiring an adequate power supply of 5 V from the MCU. A gain (A_v_) of two was set in order to double the amplitude of the fetal signal. To remove the high noise attenuation of the amplified signal, two second-order Sallen Key filters were cascaded together to form a fourth-order low-pass Butterworth filter owing to its steep filter response. This allows the signals with fc > 200 Hz to be attenuated at a higher magnitude, allowing the retrieval of just the fetal heart sound at an intensity > 60 dB. To accomplish this, the LM741 (Texas Instruments, Dallas, TX, USA), which is well-suited to the required specifications such as low cost, free availability, and the required Gain Bandwidth, (GBW) ≥ 100 × 200 (fc) = 20 kHz, was chosen. To ensure the proper functioning of the amplification and filtration circuit, a simulation was carried out using Proteus© Software (v.8.0) [32], as shown in Figure 4 below.

**Figure 5:**
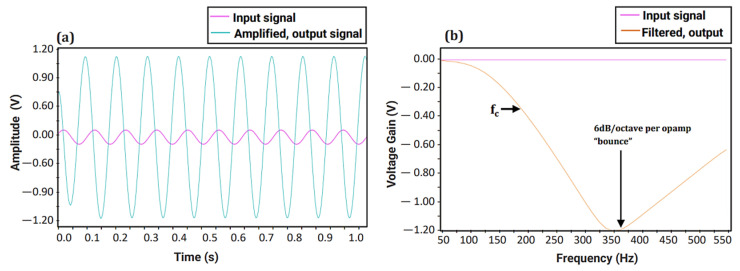
Simulation of fetal phonocardiography (fPCG) circuit: (**a**) Time domain simulation of the preamplifier and (**b**) Frequency response of designed fourth-order Butterworth Low-pass Filter (LPF).

A sine wave with an amplitude of 100 mV and frequency of 10 Hz was generated and used as the input signal. Figure 5a illustrates the time domain simulation of the preamplifier, where the input signal is amplified with a 60 dB gain, resulting in an output signal of 100 V. Figure 5b shows the response of the fourth-order Butterworth Low-Pass Filter (LPF), with a steep frequency curve and an almost ideal fc at 200 Hz. As both the filter and amplifier show the desired amplification and steep frequency response, the two circuits were implemented as one to acquire fetal heart sounds for FHR monitoring.

**Figure 6:**
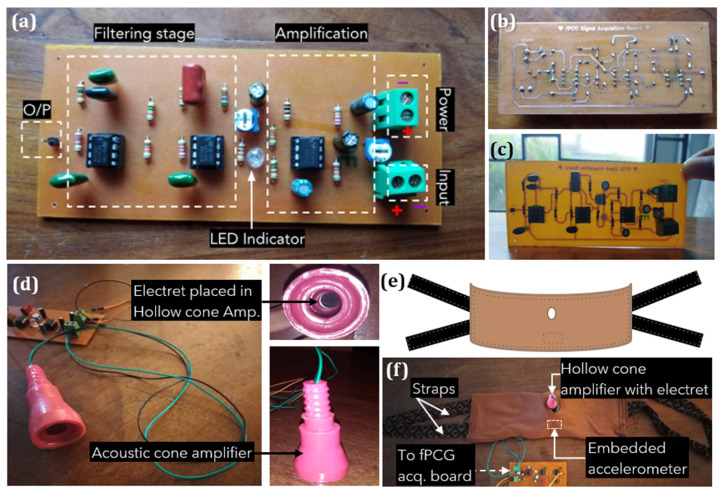
Overall system configuration of non-intrusive wearable prototype for instant home fetal movement and distress detection: (**a**) Built fetal phonocardiography (fPCG) signal acquisition board, (**b**) Soldered, back view, (**c**) Front component view, (**d**) Designed acoustic, hollow cone amplifier with an electret microphone, (**e**) Design of maternal belt and (**f**) Designed maternal belt with embedded sensors and cone amplifier attached.

The first step of device hardware implementation involved building the amplification and filter unit for fPCG signal acquisition, as shown in Figure 6. The circuit was drawn using an online design tool, then etched into a Printed Circuit Board (PCB), and components soldered to complete the PCB. Figure 6a illustrates the assembled fPCG signal acquisition board with labeled filtering and amplification stages, terminal blocks for power input, condenser microphone input, and the filtered and amplified output via a single male header pin. The input from the electret condenser microphone is first amplified with the NE5532 op-amp circuit. Subsequently, the amplified output is sent via a 10 k potentiometer to the fourth-order low-pass filter circuit employing two LM741 op-amps. The output of the filter can then be connected to the analog input pin of the MCU. The choice of components and design was driven by a commitment to cost-effectiveness. The reasoning behind selecting to develop an analog fPCG signal acquisition board stemmed from its capacity to reliably capture fetal signals while maintaining low costs. By utilizing inexpensive and readily available components, such as resistors, capacitors, operational amplifiers NE5532, LM741 ICs, and an electret condenser microphone, the system ensures efficient monitoring of fetal heart rhythm without compromising performance.

Figure 6d depicts a hollow cone amplifier that mimics the curvature of the Pinard fetoscope. Due to the variety of sounds picked up by the microphone, such as external ambient noise and distortion caused by maternal movement artifacts, the inner dimensions of the acoustic cone, which fits the electret, are used for passive amplification of the fPCG signal. A comfortable, wide elastic belt was fabricated using ultra-soft Jersey-knit material chosen for its non-allergenic, breathable, and elastic properties. The belt accommodates various body sizes, maintaining sensor positions with an embedded accelerometer and condenser on rubber foam. Straps made of soft cotton, hand-sewn on either side, secure the belt around the abdomen for a proper fit.

### 2.4. Development of the Alarm System

To implement the alarm system, a 3.7 V 1000 mAh lithium battery was used as an external power supply for the compact SIM800L GSM module, which runs on 3.7–4 V. The use of the SIM800L GSM module not only enhanced cost efficiency, primarily due to its low cost and reduced power consumption, but also enabled a more compact, economical system design. To maintain a stable power supply during transmission bursts, a 16 V 2200 μF electrolytic capacitor was used. Additionally, the use of a PCB antenna allowed for uninterrupted transmission when encased. In regard to the innovative aspects of the system, the integration of the GSM alert system, which immediately notifies the mother upon detecting fetal distress, is a feature lacking in most existing devices. This, combined with the other components of the system, provides a comprehensive approach to both monitoring and alerting for fetal distress, potentially preventing fetal death through timely medical intervention. To implement text messages, a SIM card was inserted into the module and connected to the MCU via the receiver and transmission pins. A custom-coded function sends alert messages to proxy contacts and EMS when fetal distress symptoms are detected based on FHR and movement count. For displaying FHR and FM count, an I2C module was interfaced with the 16 × 2 LCD for a faster data display rate. This reduces the number of pins required and connects to the SDA and SCL pins of the MCU. To connect the I2C bus of the MCU for serial display, the I2C address was first determined. Three 5 mm LEDs were used to indicate different warning signs: Green—Reassuring fetal status, Yellow—Deteriorating fetal condition (Low FM count), and Red—Fetal Distress (Emergency status).

## 3. Results and Discussion

### 3.1. Digital Signal Processing Algorithm for Calculating Fetal Movement

#### 3.1.1. Fetal Movement Dataset

In order to implement digital signal processing of the FM signal, an open-source dataset from the Zenodo research repository, recorded by Charlier et al. [26], was used. The dataset contains signals from 16 pregnant mothers (Gestational week not specified), acquired from a single ADXL355 (Analog Devices Inc., Norwood, MA, USA) accelerometer placed on the mother’s abdomen, with a sampling frequency of 500 Hz. Along with signal data, it also contains push-button markers for maternal perception of fetal movement. From the available data, five signal sets were selected for a feasibility test. The statistical analysis of these raw signals is presented in Table 2 below.

Table 2 presents the mean acceleration values along with the corresponding standard deviations derived from all five selected records in the publicly available database. The sample sizes for each record are provided, with Record 1 containing the fewest samples (154,100) and the shortest corresponding time length of 5.14 min, while Record 5 includes the highest number of points sampled (451,421) and the longest duration of 15.05 min. The standard deviation values in Table 2 indicate the variability of the combined signals of fetal movement, maternal artifacts, and noise in each axis direction. In all five records, the Y-axis shows the highest variability. The standard deviation is lowest on the X-axis for two records but lowest on the Z-axis for three records.

#### 3.1.2. Fetal Movement (FM) Signal Visualization and Filtering

**Figure 7:**
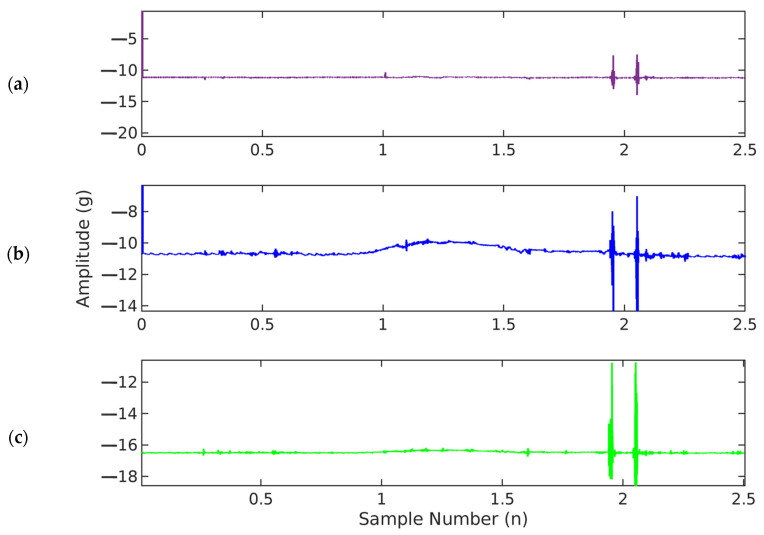
Graphical illustration of fetal accelerometer movement data (fs = 500 Hz) in (**a**) X-axis, (**b**) Y-axis, and (**c**) Z-axis.

From the dataset, a single record containing accelerometer data from all three axes was loaded into MATLAB. The accelerometer measures fetal movement accelerations along all three axes. The X and Y axes record acceleration along the plane of the maternal abdomen, while accelerations normal to the maternal abdomen are recorded in the Z-axis (Figure 7). The vertical axis shows the amplitude of acceleration, recorded in gravitational acceleration units (g), while the horizontal axis shows the time in seconds (s).

Next, the accelerometer data was fused to obtain one signal containing information from all three axes by using Equation (1) below.

(1) $$A_{xyz}=\sqrt{{\left(A_{x}\right)}^{2}+{\left(A_{y}\right)}^{2}+{\left(A_{z}\right)}^{2}}$$

**Figure 8:**
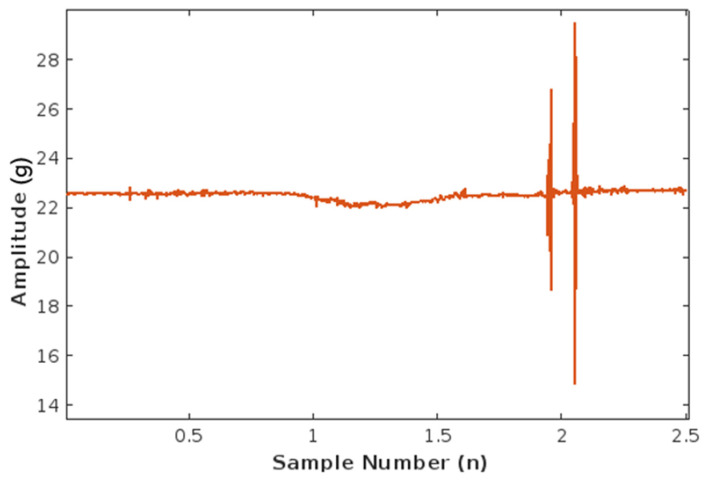
Plot of fetal movement accelerometer data (fs = 500 Hz) fused into one.

The horizontal axis now contains the discrete sample number *n*, which is consistent with the sampling frequency of 500 samples per second. The Nyquist sampling theorem was followed during the data acquisition to prevent signal aliasing by reliably converting an analog signal in continuous time, t, to a discrete set of values, *n*. The resulting plot of the fused signal is illustrated in Figure 8.

**Figure 9:**
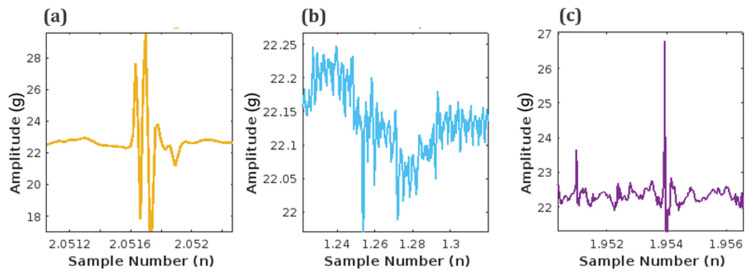
Artifacts embedded in acquired unfiltered fetal signal: (**a**) Maternal laughter, (**b**) Maternal respiration, and (**c**) Fetal movement (FM) signal.

The unfiltered signal contains a significant amount of noise and maternal signal artifacts. Maternal laughter and respiration are the most prominent among maternal signal artifacts. Figure 9 shows the different time stamps at which these three signals occur during the length of the unfiltered signal in Figure 8.

Filtering was performed using a high-order infinite impulse response (IIR) or Notch filter to remove the high-frequency artifacts pictured in Figure 9, such as maternal laughter, which can be greater or similar in amplitude to fetal movement and low-frequency breathing movements while extracting the FM signal. The following digital filter transfer function listed in Equation (2) was used for this purpose:

(2) $$h\left(n\right)=\frac{1}{T}\left[\frac{1-Z^{-1}}{1-0.99Z^{-1}}\right]$$

**Figure 10:**
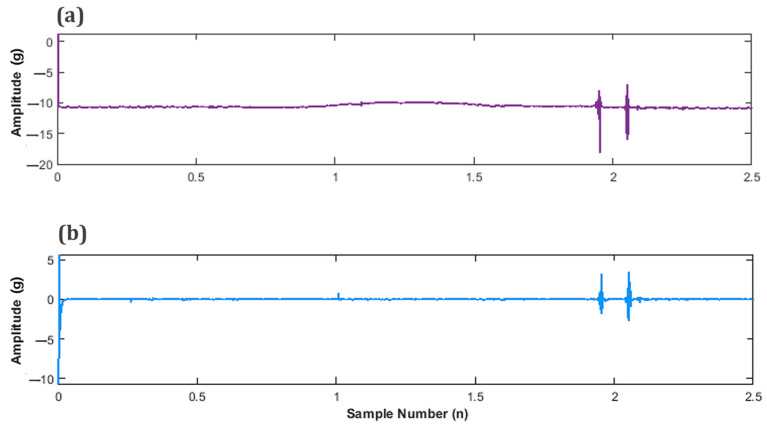
Subplots of fetal movement (FM) accelerometer data (**a**) Unfiltered FM signal and (**b**) Filtered, detrended FM signal.

Figure 10a displays the unfiltered signal, providing a point of comparison for the successfully detrended signal illustrated in Figure 10b. 

#### 3.1.3. Fetal Movement (FM) Peak Detection

**Figure 11:**
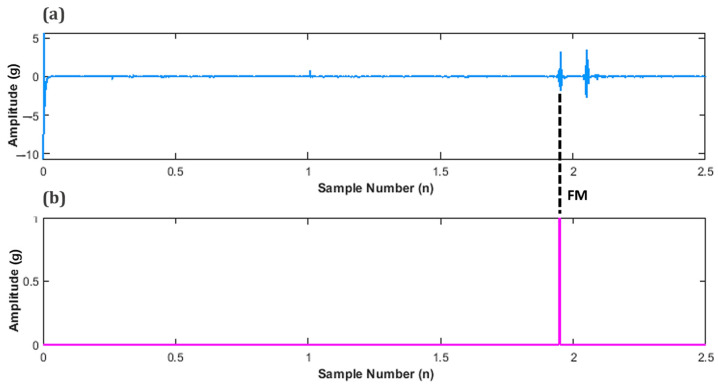
Subplots of fetal movement (FM) accelerometer data (**a**) Unfiltered FM signal, and (**b**) Maternal perception of FM.

In order to determine the peak value of FM, the values for maternal perception were sub plotted in-line, below the filtered signal as shown in Figure 11. This was performed to determine the instance of an FM peak and its corresponding amplitude.

This concept can be represented by the following Equation (3)

(3) $$P_{FM}=|\left\{t\;|X_{filt}\left(t\right)>T\right\}|$$

where $P_{FM}$ = Number of FM peaks detected, *t* = time, $X_{filt}\left(t\right)$ = filtered fetal signal at time (*t*) and *T* = threshold value.

**Figure 12:**
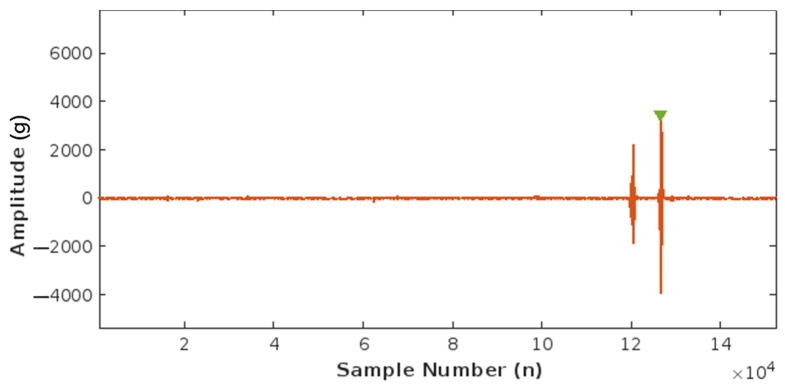
Plot of Fetal Movement (FM) detection corresponding to one FM peak.

Using this correlation, an FM peak value of around 0.015–0.002 g was found and set as the peak detection threshold to count fetal movements. Figure 12 below illustrates the detection of one FM peak via the threshold method.

This acquired value of 0.002 g is an estimation based on the data available. To accurately calibrate the proposed device, clinical trials must be conducted to monitor fetal signals during the second and third trimesters of pregnancy which necessitates ethical clearance and approval.

#### 3.1.4. Fetal Movement (FM) Count Algorithm

**Figure 13:**
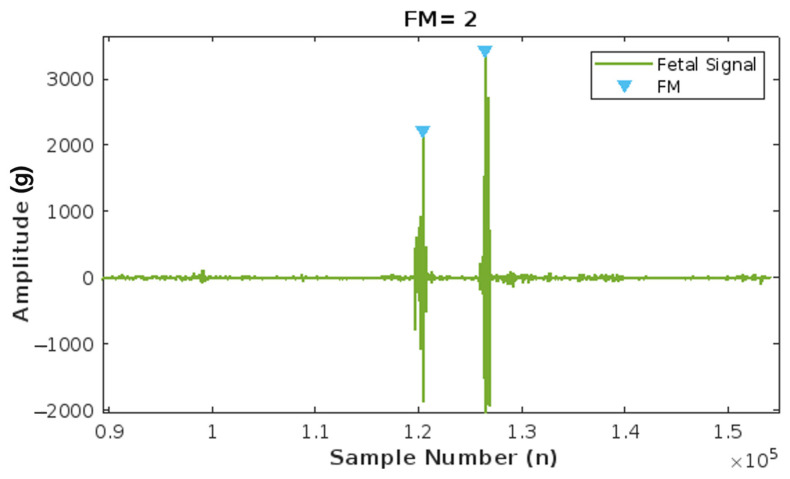
Results of the Fetal Movement (FM) count algorithm, with two peaks corresponding to two FMs.

The FM count algorithm was tested by running the algorithm with five different accelerometer sample data taken from the same database by Charlier et al. [30] used for its implementation. Figure 13 illustrates the number of fetal movements detected by the algorithm. The data are loaded, and upon fusion, detrending, and filtering of the recorded signal, the amplitude is measured. If the amplitude of the fetal signal exceeds a threshold of 0.002 g, one fetal movement is counted. The graph visually represents how two major peaks, each with an amplitude greater than 0.002 g, are recorded as two fetal movements (FM), demonstrating the algorithm’s effectiveness.

Using the same method, four other fetal movement records are subsequently loaded, and the number of FMs counted by the algorithm is recorded. The results were validated by holding maternal perception as the ground truth and comparing the recorded values (Figure 13). Maternal perception of FM is determined by asking the mother to click on a button every time she perceives a fetal movement. This input is logged alongside the fetal accelerometer data within the same time frame. 

**Figure 14:**
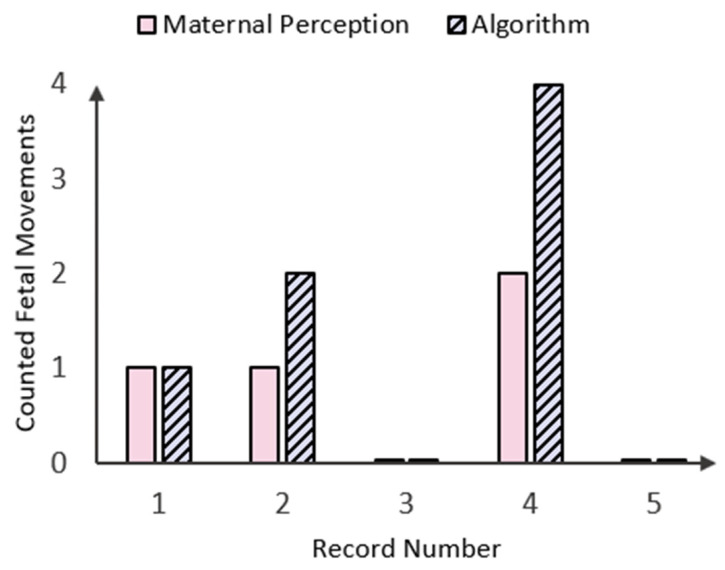
Bar graph displaying the number of fetal movements (FMs) perceived by the mother in comparison to those counted by the peak detection algorithm in five patient records.

As shown in the bar chart in Figure 14 above, there were three instances where fetal movements counted by both maternal perception (MP) and the algorithm aligned. Specifically, one fetal movement was recorded in Record Number 1, and no fetal movements were observed in Record Numbers 3 and 5. However, there were two instances concerning Record Numbers 2 and 4, where the algorithm detected twice as many fetal movements while maternal perception (MP) declared only one and two FMs, respectively. This could either be due to the small time window between two instantaneous fetal kicks, which the mother perceived as one FM, or it could be the occurrence of maternal laugh, which lies in the same amplitude as the FM signal (Figure 9). In a similar study conducted by Delay et. al. [15], maternal laughter was accounted for by recording inputs via a separate button system, but as the accelerometer data used in this study were taken from an open-source database, there was no way of identifying when maternal laughter occurred. To address these limitations, the device should be calibrated with real-time data from pregnant mothers, incorporating variables like push-and-hold maternal perception methods, push-button markers for maternal movements (e.g., laughter, coughing, and hiccups), and extended recording durations. Additionally, the device could be simulated over longer periods (e.g., 20–30 min) and compared with benchmark methods such as fECG and clinical ultrasounds to further validate its performance. 

However, as testing of the fetal movement signal processing algorithm (fmSP) was based on the assumption that the MP was the ground truth, results showed that while there was some variation in the number of FM counts, the fmSP algorithm produced zero false negative results. More comparative tests (i.e., clinical ultrasounds) must be run to determine whether the proposed device can identify fetal movements that cannot be perceived by the mother herself.

### 3.2. Amplifier and Filter for Fetal Heart Rate Acquisition

**Figure 15:**
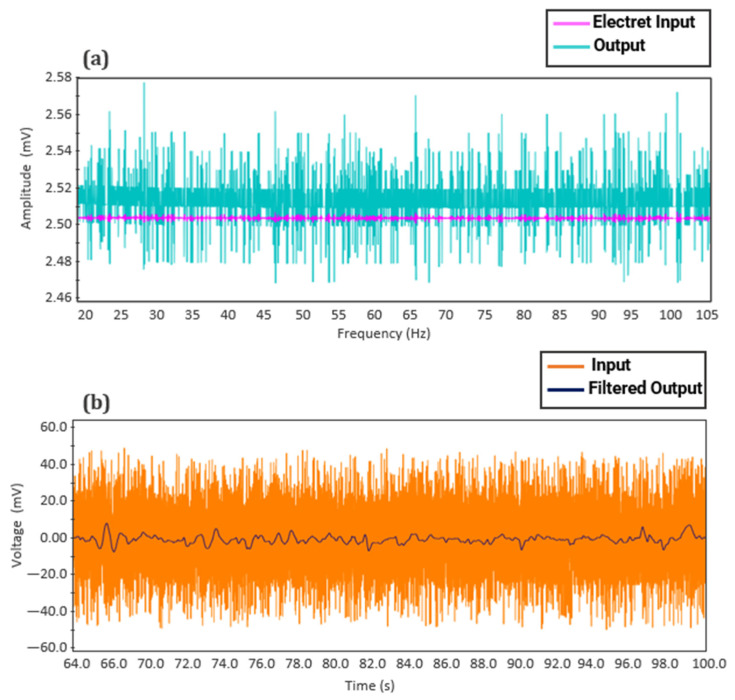
Proteus© (v.8.0.) simulation results of fetal phonocardiography (fPCG) circuit: (**a**) Pre-amplifier and (**b**) fourth-order Butterworth filter.

Testing of the fPCG acquisition board was conducted using Proteus© (v.8.0) simulation software and a heart WAV. file from an open-source media website [33] in order to see the resulting output of the designed fourth-order Butterworth filter and pre-amplifier. As illustrated in Figure 15a below, the electret input signal (from the simulated electret condenser microphone), possessing an amplitude of around 0.0025 V, has been amplified with a 60 dB gain to give an output signal nearly double the original amplitude. Figure 15b shows the amplified signal being appropriately filtered to obtain a clean fetal heart sound signal while preserving useful signal data. The peaks corresponding to the individual fetal heart rate complexes can be distinctly seen, ranging around 5.0 mv. In a similar study conducted by Ahmad et al. [34], fetal heart rate monitoring using a condenser microphone was validated and compared to standard devices, yielding consistent results.

This demonstrates the effectiveness of the simulated system in analog amplification and filtering, which enables the microcontroller to acquire a fetal signal containing all pertinent data on the current fetal status while eliminating ambient noise and motion artifacts.

### 3.3. Alarm System

Two potentiometers were utilized to simulate fetal heart rate and fetal movement by varying the voltage input to the MCU through rotation to test the alarm system. Based on the thresholds set for fetal movement (FM) and fetal heart rate (FHR), the system’s algorithm displayed the relevant information and activated the alarm system, as detailed in Table 3.

As shown in Table 3, all components of the alarm system met the expected outcomes and were successful in its overall implementation.

Many of the device’s limitations stemmed from the limited variables in the dataset used. For one, the open-source dataset did not account for occurrences such as maternal laughter, which made it challenging to assess the reason behind the doubled fetal movement counts. The functionality can be further enhanced by facilitating real-time data collection, which can provide more accurate insights into fetal movements and maternal responses. The absence of real-time monitoring limits the ability to assess the device’s performance in dynamic, real-world conditions. Additionally, the utilized dataset had relatively short recording durations, spanning from 5–15 min. This limited duration might not capture the full range of fetal movements and maternal activities considering that the average period of fetal rest, where no fetal movement is expected, lies between 22–75 min. Therefore, longer recording periods are essential to better evaluate the performance of the algorithm over extended timeframes. Moreover, the limited diversity in maternal profiles, such as maternal age, body mass index (BMI), and pregnancy conditions, as well as a lack of event varieties such as contractions, maternal exercise, and different fetal movement patterns, could impact the generalizability of the results. Therefore, testing across a wider range of maternal profiles using a more comprehensive dataset with diverse events would be necessary to ensure the effectiveness of the device across different populations. Other limitations include potential sensor calibration issues, as the dataset may not account for variations in sensor placement or calibration, which could affect the consistency and accuracy of the data collected. Proper sensor calibration is crucial to ensure reliable fetal movement and distress detection. Further, the absence of comparative benchmarking, such as comparison with established clinical methods, like ultrasound or fetal electrocardiography (fECG), poses another limitation in terms of further, more accurate validation of the device.

## 4. Conclusions

The early-stage prototype assessment of a cost-effective, non-intrusive, wearable device for instant home fetal movement and distress detection was proposed to overcome limitations in existing fetal monitoring systems, such as the emission of radiation, the need for trained medical professionals, serial clinical visits, and high costs. The use of low-cost, non-invasive sensors made the proposed device safe, less complex, and more affordable. The incorporation of a digital signal processing algorithm allowed further refining of the fetal signal, which played a vital role in accurately calculating fetal heart rate, variability, and fetal movement. Accurate calculation is important as false positives can put the mother under unnecessary stress, while false negatives can be detrimental to the health of the fetus. The results showed that while there was some variability in counted fetal movements, the proposed algorithm was successful in achieving zero false negative results. The alarm system was timely and accurate in sending alert messages upon the detection of fetal distress. Many of the device’s limitations stemmed from the limited variables in the dataset used. The open-source dataset did not account for occurrences of maternal laughter, which made it challenging to assess the reason behind the doubled fetal movement counts. To address these limitations, the device should be calibrated with real-time data from pregnant mothers, incorporating variables like push-and-hold maternal perception methods, push-button markers for maternal movements (e.g., laughter, coughing, and hiccups), and extended recording durations. Additionally, the device could be simulated over longer periods (e.g., 20–30 min) and compared with benchmark methods such as fECG and clinical ultrasounds to further validate its performance. For the further development of the device, a software application can be integrated to set custom thresholds to improve device precision in calculating FM. Calculation of baseline variability holds great clinical value in determining the long-term factors contributing to Fetal Distress. Finally, the inclusion of machine-learning classification algorithms to automatically exclude maternal artifacts and discriminate between the different types of fetal movement will further improve reliability and accuracy. In conclusion, all the proposed aims and objectives were fulfilled, potentially offering expectant mothers a sense of comfort and security, saving one fetus at a time. 

## Figures and Tables

**Table 1:** Comparison of existing fetal monitoring devices and prototypes.

Sensor and Electrode Configuration	Monitoring (Method, Sensors, Gw)	Algorithm and Signal Acquisition Techniques Used	Data Processing	Ref.
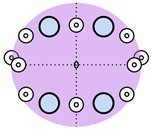	▪ fPCG ▪ Acoustic and electrical sensors ▪ FHR, UTC ▪ 30th GW	Construction of mECG template through maternal QRST peak detection and mECG cancellation. fs = 120 Hz	Offline	[14]
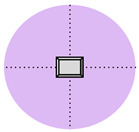	▪ fPCG ▪ Accelerometer ▪ FM ▪ 26th GW	Hybrid algorithm (High pass filtering, segmentation, STFT, with CNN architecture) fs = 280 Hz	Offline	[15]
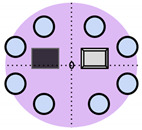	▪ fPCG ▪ Acoustic sensors, accelerometer, and IMU ▪ FM ▪ 25 to 35 + 6th GW	Segmentation (Comb-notch filtering), Signal matching and Discrimination using PCA.	Offline (IMU and analog data on SD card)	[16]
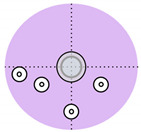	▪ fECG ▪ Biopotential electrodes ▪ FHR	Baseline drift and mean value cancellation. FastICA algorithm (fECG extraction), sample entropy (signal location). fs = 250 Hz	Real-time via Bluetooth and Cloud storage	[17]
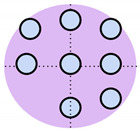	▪ fPPG ▪ Optical fiber sensors ▪ FM ▪ 28th and 34th GW	Fast ICA algorithm (removes breathing artifacts and extract fECG).	Offline	[18]
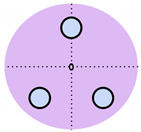	▪ fSGC and fGCG- ▪ Gyroscope and accelerometers ▪ FHR ▪ 40th GW	Prefiltered with zero-phase IIR filter CWT for processing and averaging algorithm for fusion. fs = 250 Hz	Offline	[19]
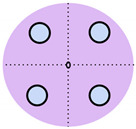	▪ fPCG ▪ piezoelectric vibration sensors ▪ FHR ▪ 30–40th GW	Decomposition technique using Eigen vectors (to separate into different sound components) Denoising using WTST-NST. fs = 100 Hz	Offline	[20]
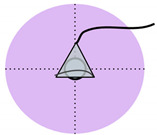	▪ fPCG ▪ condenser microphone ▪ FHR	Butterworth filter for sharpening sound attenuation in analog pre-amplification process.	Offline	[21]

**Table 2:** Statistical analysis of open-source fetal movement accelerometer data |*n* = 5|fs = 500 Hz.

Record Number (*n*)	Axis	Mean Value	Standard Deviation	Number of Samples (*n*)	Total Time Length (min)
Record 1	X	−11.1521	0.0706	154,100	5.14
	Y	−10.5529	0.2797		
	Z	−16.4638	0.0880		
Record 2	X	−11.8295	0.0521	450,663	15.02
	Y	−10.5699	0.0983		
	Z	−16.4497	0.0324		
Record 3	X	−11.0093	0.0190	451,049	15.03
	Y	−9.3378	0.0417		
	Z	−16.1127	0.0302		
Record 4	X	−13.5053	0.1141	451,421	15.05
	Y	−10.2576	0.0671		
	Z	−15.9186	0.0600		
Record 5	X	−10.2336	0.0705	451,609	15.05
	Y	−10.3554	0.0720		
	Z	−16.3136	0.0547		

**Table 3:** Summary of initiation and alarm system testing results.

Components	Variable	Expected Results	Final Outcome
Initiation	Initialization of system	White LED ON, LCD displays “Initialising”	White LED ON Displays: “Fetal Monitor Initialising…”
Alarm System	Reassuring Fetal Status	Green LED ON FHR and FM within normal range.	FHR = 140 BPM FM = ACT Displays: “Normal” Green LED ON
	Low Fetal Movement count	Yellow LED On Warning Text: Low FM Count	FHR = 140 BPM FM = LOW Displays: “Monitor” Texts to Proxy: “Warning: Low Fetal Movement count” Yellow LED ON
	Non reassuring fetal status	Red LED ON FHR and FM outside normal range: Fetal distress	FHR = 162 BPM or 116 BPM FM= ACT or LOW Displays: “! Fetal Distress!” Texts to Proxy: “EMERGENCY: Fetal Distress Detected!” Red LED ON

## Data Availability

The original contributions presented in the study are included in the article, further inquiries can be directed to the corresponding authors.
